# Supplementary material for: Recommending Physical Activity for People with Intellectual Disabilities: The Relevance of Public Health Guidelines, Physical Activity Behaviour and Type of Contact
Source: Int J Environ Res Public Health. 2023 Apr 17;20(8):5544. doi: 10.3390/ijerph20085544 (PMC10138360; doi:10.3390/ijerph20085544)
Supplement: Supplementary file 1 [file ijerph-20-05544-s001.zip › ijerph-2234206-supplementary.pdf]

By participating, you agree that your anonymously collected data may be used in the course of the scientific survey of this study. The data will not be passed on to third parties in the sense of the GDPR and will be used exclusively for research-related purposes. For more information on data protection, please click the policy below.

Section A: Points of contact to people with intellectual disability

"Intellectual disability" in this questionnaire refers to impairments in development, learning, or social behavior such as in trisomy 21 (Down syndrome), autism spectrum disorders, or learning difficulties associated with intelligence impairment.

A1. Do you actually have direct contact with people with intellectual disabilitiy?

Yes ☐

No ☐

A2. Have you had direct contact with people with intellectual disability in the past?

Yes ☐

No ☐

A3. In which context and how often do you have or had contact with people with intellectual disability?

	never - not at all	rarely - once a month	sometimes - once per week	frequently - several times a week
Family	☐	☐	☐	☐
Circle of friends	☐	☐	☐	☐
Study	☐	☐	☐	☐
Work	☐	☐	☐	☐
Neighborhood	☐	☐	☐	☐
Sports club	☐	☐	☐	☐
Other club / organization (without sports orientation)	☐	☐	☐	☐
Other leisure time (not club/organization related)	☐	☐	☐	☐
Others	☐	☐	☐	☐

A4. Which?

A5. How would you describe the closeness of your contacts to people with intellectual disability in general?

not close at all ☐ ☐ ☐ ☐ ☐ very close

A6. How physically active are the individuals with intellectual disability you just thought of?

Not at all active ☐ ☐ ☐ ☐ ☐ Very active

Section B: Intellectual disability

"Intellectual disability" in this questionnaire refers to impairments in development, learning, or social behavior such as in trisomy 21 (Down syndrome), autism spectrum disorders, or learning difficulties associated with intelligence impairment.

B1. How well would you rate your knowledge of intellectual disability in general?

not good at all ☐ ☐ ☐ ☐ ☐ very good

B2. How would you rate your own ability in dealing with people with intellectual disability based on the following questions?

	not at all true				completely true
I can rely on my skills in challenging situations.	☐	☐	☐	☐	☐
I am convinced that I can maintain an appreciative relationship.	☐	☐	☐	☐	☐
I can cope well with any difficulties that may arise under my own steam.	☐	☐	☐	☐	☐
Generally speaking, I find encounters with different people enriching.	☐	☐	☐	☐	☐
I can usually solve even demanding and complicated tasks well.	☐	☐	☐	☐	☐

Section C: Recommendations

The use of the term "physical activity" means all activities in everyday life that stimulate energy consumption, with different intensities:

Low intensity is hardly perceived as effort (e.g. going for a walk). **Moderate intensity** is defined as a noticeable increase in pulse and respiratory rate, with conversation still easily possible during the activity (e.g. Nordic Walking). **High intensity** is defined as a significant increase in pulse and respiratory rate, where continuous conversation is no longer possible (e.g. fast running).

C1. Would you recommend physical activity to people with intellectual disability (without physical impairment)?

Yes ☐

No ☐

C2. **How much time and intensity for physical activity per week would you recommend to people with intellectual disability (without physical disability)? Low intensity physical activity: slight increase in pulse and respiratory rate, conversation possible at any time during activity**

Minutes per week ☐☐☐

C3. **How much time and intensity for physical activity per week would you recommend to people with intellectual disability (without physical disability)? Physical activity with medium intensity: noticeable increase in pulse and respiratory rate, conversation still possible during activity**

Minutes per week ☐☐☐

C4. **How much time and intensity for physical activity per week would you recommend to people with intellectual disability (without physical disability)? High intensity physical activity: noticeable increase in pulse and respiratory rate, difficult to talk during activity**

Minutes per week ☐☐☐

C5. **Would you recommend muscle-strengthening exercises such as push-ups, sit-ups, exercises with Thera-Band or dumbbells, or specific yoga exercises?**

Yes ☐

No ☐

C6. **How many days per week would you recommend such exercises?** ☐

C7. **Please give brief reasons for your recommendations:**

C8. **What are your recommendations based on?**

Own experience ☐

Own sport behavior ☐

Own experience in dealing with people with intellectual disability ☐

Exercise recommendations or other documents ☐

Knowledge from a training or further training course ☐

Other ☐

Other

C9. Did the constraints around the Covid pandemic influence your response regarding the recommendation?

Yes ☐

No ☐

C10. Please briefly justify your choice:

Section D: Sedentary behavior

D1. To what extent would you classify long periods of sitting (sedentary behavior) in people with intellectual disability (without physical impairment) as a health risk?

not high at all	rather not high	neither	rather high	very high
☐	☐	☐	☐	☐

Section E:

E1. Which of the following documents indicating the relevance of exercise and physical activity are you familiar with?

	do not know	know parts of the content	know the content approximately	know the most important parts of the content	know all the content
German physical activity recommendations from 2016	☐	☐	☐	☐	☐
Austrian physical activity recommendations from 2020	☐	☐	☐	☐	☐
Swiss physical activity recommendations from 2017	☐	☐	☐	☐	☐
Physical activity recommendations of the WHO from 2020	☐	☐	☐	☐	☐
UN Convention on the Rights of Persons with Disabilities [UN CRPD] (in particular Article 30)	☐	☐	☐	☐	☐

E2. **Are you aware of any other documents?**

- Yes ☐
- No ☐

E3. **Which other documents are you aware of?**

E4.

	do not know	know parts of the content	know the content approximatel y	know the most important parts of the content	know all the content
How well do you know them?	☐	☐	☐	☐	☐

Section F:

F1. **What media and channels do you use to learn about physical activity and exercise recommendations?**

- Printed brochures ☐
- Books ☐
- Professional articles and journals ☐
- Internet ☐
- Specialized organizations ☐
- None ☐
- Other ☐

Other

Section G: Own physical activity behavior

The questions will ask you about the time you spent being physically active in the **last 7 days**. Please answer each question even if you do not consider yourself to be an active person. Please think about the activities you do at work, as part of your house and yard work, to get from place to place, and in your spare time for recreation, exercise or sport.

G1. **Think about all the vigorous activities that you did in the last 7 days. Think *only* about those physical activities that you did for at least 10 minutes at a time. During the last 7 days, on how many days did you do vigorous physical activities like heavy lifting, digging, aerobics, or fast bicycling?**

***Vigorous** physical activities refer to activities that take hard physical effort and make you breathe much harder than normal.*

☐

G2. **How much time did you usually spend doing vigorous physical activities on one of those days? (in minutes)**

☐☐☐

G3. **Think about all the moderate activities that you did in the last 7 days. Think only about those physical activities that you did for at least 10 minutes at a time. During the last 7 days, on how many days did you do moderate physical activities like carrying light loads, bicycling at a regular pace, or doubles tennis? Do not include walking.**

***Moderate** activities refer to activities that take moderate physical effort and make you breathe somewhat harder than normal.*

☐

G4. **How much time did you usually spend doing moderate physical activities on one of those days?**

☐☐☐

G5. **Think about the time you spent walking in the last 7 days. During the last 7 days, on how many days did you walk for at least 10 minutes at a time?**

This includes at work and at home, walking to travel from place to place, and any other walking that you have done solely for recreation, sport, exercise, or leisure.

☐

G6. **How much time did you usually spend walking on one of those days?**

☐☐☐

G7. **The last question is about the time you spent sitting on weekdays during the last 7 days. During the last 7 days, how much time did you spend sitting on a week day?**

Include time spent at work, at home, while doing course work and during leisure time. This may include time spent sitting at a desk, visiting friends, reading, or sitting or lying down to watch television.

☐☐☐

G8. **Has your own physical activity changed due to the restrictions related to the Covid pandemic?**

Yes ☐

No ☐

G9. **How has your physical activity changed due to the restrictions related to the Covid pandemic?**

	less physical active	same activity	more physical active
	☐	☐	☐
	☐	☐	☐
	☐	☐	☐

G10. **Please briefly explain your choice**

Section H: Sociodemographic data

Finally, we ask you to provide a few socio-demographic data.

H1. **Please let us at the beginning know in which country you are currently located?**

- Germany ☐
- Austria ☐
- Switzerland ☐
- Liechtenstein ☐
- Other ☐

Other

H2. **Which gender do you feel you belong to?**

- female ☐
- male ☐
- other ☐

H3. **Age**

H4. Which size has your place of residence?

- less than 5,000 inhabitants ☐
- 5,000 to 10,000 inhabitants ☐
- 10,000 to 50,000 inhabitants ☐
- 50,000 to 100,000 inhabitants ☐
- 100,000 to 1,000,000 inhabitants ☐
- more than 1,000,000 inhabitants ☐

H5. What is the size of your place of work?

- less than 5,000 inhabitants ☐
- 5,000 to 10,000 inhabitants ☐
- 10,000 to 50,000 inhabitants ☐
- 50,000 to 100,000 inhabitants ☐
- 100,000 to 1,000,000 inhabitants ☐
- more than 1,000,000 inhabitants ☐

H6. What is your current job?

H7. Highest level of education completed:

- No education, primary (school-leaving certificate and "apprenticeship") ☐
- Secondary (e.g. vocational training) ☐
- Tertiary (e.g. UNI, UAS, etc.) ☐

H8. Did your training involve people with disabilities?

- Yes ☐
- No ☐

H9. Were topics on exercise and sport part of your training?

- Yes ☐
- No ☐

H10. What is your general work-related experience (in years)?

H11. How would you rate your digital literacy (e.g. safe and critical use of digital technologies)?

not good at all ☐ ☐ ☐ ☐ ☐ very good

H12. Do have a sports related qualification?

- None ☐
- Study of sports science ☐
- D-Trainer / exercise instructor ☐
- C-Trainer / Instructor ☐
- B-Trainer / Teacher ☐
- A- Trainer / Trainer ☐
- Esa / J + S leader ☐
- Esa / J + S expert ☐
- Assistance (module) in sports for the disabled ☐
- Disabled sports instructor ☐
- Disability specific training ☐
- Snow sports instructor ☐
- Teacher ☐
- Study of special education needs ☐
- Other ☐

Other

[ ]

H13. At the end, we ask you to enter a code to ensure anonymity and assignment in case of questions on your part or a possible later follow-up survey. This consists of the first and last letter of your mother's first name, your father's first name and the date of your mother's birth. Your CODE:

Here is an example: First name mother: ANNE, First name father: THORSTEN, Date of birth mother: 17 July 1955

This results in a code: ***A E T N 1 7***

H14. Is there anything else you would like to share on this topic?
